# Virolithopanspermia: Might viruses be transported in rocks through space?

**DOI:** 10.1371/journal.ppat.1012955

**Published:** 2025-03-18

**Authors:** Frederic D. Bushman

**Affiliations:** Department of Microbiology, Perelman School of Medicine, University of Pennsylvania, Philadelphia, Pennsylvania, United States of America; Mount Sinai School of Medicine, UNITED STATES OF AMERICA

## What is virolithopanspermia?

Material can apparently move between planets, at least on rare occasions. For example, meteorites have been found on Earth that appear to have originated on Mars [1], or even outside our solar system [2]. The theory of panspermia posits that life might move between planets, and panspermia may be the origin of life on Earth.

However, the process of launching material off one planet and then passing through the atmosphere to land on another is harsh, likely killing off most living material transferred in the process. This has led to the conjecture that if panspermia happens, it might involve cells or spores protected inside rocks. Such protective packaging would diminish exposure to damaging heat, radiation, and cosmic rays. Thus, “lithopanspermia” is the idea that cellular life may move between planets inside rocks [3].

But as is described below, for any rock on Earth hosting cellular life, it will commonly host even more viral particles. This essay thus explores the idea of “virolithopanspermia”, the transport of viral particles between planets in rocks. It seems plausible to speculate that Earth may be spraying viruses into space at a low rate, and going the other way, that extraterrestrial material landing on Earth may on occasion contain recognizable viral particles.

## How abundant are viruses on Earth’s surface, and how well can viruses survive extreme conditions?

Oceans cover ~70% of Earth’s surface, so that a meteor hitting Earth would likely hit in an ocean. Prokaryotic cells can be highly abundant in sea water, up to 10^6^ bacteria per ml; remarkably, they are commonly outnumbered by their viruses (“bacteriophage” or “phage”), which can reach 10^7^ per ml [4, 5]. Viruses and cells can settle to the ocean floor, so that beneath the oceans, a deep microbial biosphere extends hundreds to thousands of meters down in marine sediments. Early studies have also documented the presence of an enormous virosphere [6]. Prokaryotic cells predominate in the deep marine sediments, and are thought to number fully 3 × 10^29^ cells globally, accounting for around half of all the prokaryotic cells in the ocean [6, 7]. Viruses in marine sediments may number up to 1.8 × 10^10^ viruses per cubic centimeter [6], potentially outnumbering their hosts.

Sequences from bacterial viruses have been reported in samples taken from a 2.5-km deep drill hole in Finland [8], a 2.5-km deep shale fracture system in the Appalachian basin [9] and a 3-km deep groundwater sample in South Africa [10]. In deep marine sediments, cells were fewer, but viral particle counts were relatively higher. This is potentially consistent with superior preservation of viruses at greater depths over long time periods. A possible mechanism for greater viral preservation is adherence to clays, which have higher surface charges and smaller pore spaces in deep sediments than in surface layers, potentially binding viruses and shielding them from degradation [6,11]. Metagenomic studies of glacial ice [12] and many terrestrial surface environments have also yielded rich viral populations [13, 14].

Evaluating the plausibility of virolithopanspermia turns in part on understanding how well viruses will survive in harsh environments. Many types of viruses are quite labile, and are expected to have short half-lives under unfavorable circumstances. Membrane enclosed viruses are inactivated rapidly under conditions damaging to membrane integrity [15]. At the other extreme, some nonenveloped viruses have quite tough capsids—for example, phage T1 particles can survive drying [16], and the phage HK97 capsid is actually covalently crosslinked to form tough molecular chain mail [17]. Viruses with particularly stable particles are top candidates for participating in virolithopanspermia.

## How frequently are chunks of rock blasted off Earth’s surface by impacts?

Material may be ejected from Earth into space during impacts of large meteors. Such impacts were most common early during Earth’s history in the “period of heavy bombardment”, estimated to be from 4.1 to 3.8 billion years ago [18]. The timing of the origin of cellular life is uncertain, but estimated to be from 3.5 to 4.3 billion years ago [19,20], potentially overlapping the period of heavy bombardment.

But, when did viruses arise? Recent computational and theoretical work suggests that replication systems will give rise to parasites inevitably, and phylogenetic reconstructions based on sequence data suggest that viruses have arisen independently at least six times on Earth [21]. These and other observations support the idea that viruses arose quite soon after the origin of cellular life. Thus, it seems likely that viruses also would have been present on Earth overlapping the period of heavy bombardment.

What becomes of the material blasted off Earth? Quantitative models suggest that fragments expelled from Earth experience a variety of fates. Some fall back on Earth, and some fall into the Sun. Less commonly, material reaches the other planets in our solar system, and on rare occasions even progresses out of the solar system [3,18]. Estimates suggest that over the last 3.5 billion years, approximately 3 × 10^8^ fragments have been ejected from Earth that have the potential to transfer biological materials [18].

## How likely is virolithopanspermia?

Given that viruses often outnumber their hosts in sediments and rock [6,11], it is likely that viruses on Earth will occasionally be enclosed in ejected material and launched into space during impacts. As mentioned above, viruses may be concentrated and protected in pores in deep marine clays, potentially encapsulating and shielding viruses during ejection [6,11]. It is unknown how many viral particles will retain their structure and biological activities through these processes, but given the extreme stability of some viral particles and the very large numbers of viral particles on Earth, it seems likely that at least a few may survive.

Thus, it seems reasonable to speculate that (1) viruses arose on Earth at around the time of the origin of cellular life, (2) Earth has been spraying viruses out into the solar system at a low rate during bombardments, and 3) some of this material even progresses on into interstellar space. In ejected material, viruses will commonly be more abundant than their host cells.

## Might virus-like particles be useful targets in searches for evidence of extraterrestrial life?

Viruses ejected from Earth would be unlikely to find a suitable host cell in space or on another planet, and so probably could not replicate. However, going the other way, there is great interest in investigating extraterrestrial materials for signs of life. Rocks believed to have originated on other planets and even outside our solar system have been reported, and a fascinating question centers on how best to interrogate such materials for signs of life. Virus-like particles might provide a useful analytical target.

Virus-like particles might be recognizable in extra-terrestrial rock as small assemblies of macromolecules with repetitive structural components and an internal informational polymer. Advanced imaging or mass spectrometry methods might be used to interrogate candidate particles in such rocks. A challenge is that in the analysis, one would not want to assume that, as on Earth, the informational polymer was nucleic acid, or that the capsid was composed of protein. Despite this, it might be possible to look for symmetries in viral construction that would likely be universal, and recognizable in extra-terrestrial materials.

All viruses are presented with the problem of how to enclose an informational polymer in a protective shell. Coding space is commonly limited in viral genomes, so identical capsid monomers are usually used repeatedly to construct the capsid, thereby conserving genomic space encoding capsid components. This imposes specific symmetries on particle construction. Some viruses are helical in structure, and so comprised of an open tubular structure; such an arrangement is not limited to any specific number of capsid monomers. However, another large class of viral particles are spherical, and these are constrained to construction principals based on the Platonic Solids, which are universal and involve defined numbers of subunits.

Geometric analysis going back to the ancient Greeks established that there are five and only five ways of enclosing three-dimensional space with a two-dimensional subunit. These are the tetrahedron, cube, octahedron, dodecahedron, and icosahedron. Spherical non-enveloped viruses commonly show icosahedral symmetry, which involves assembly of 20 triangular faces to form an enclosed structure. Of the five Platonic Solids, icosahedra most efficiently enclose space, and icosahedra are relatively rigid, explaining their wide occurrence in viral structures. Often each triangular face is composed of three subunits, for a total of 60 capsid subunits in a simple icosahedral particle.

However, a larger genome may require encapsidation in a larger capsid shell. Classic studies of virus structure have specified assemblies in which the capsid proteins assume near-identical conformations in larger icosahedral structures. These are comprised of specific allowable numbers of subunits, which are defined by the *T* numbers [22]. A *T* = 3 capsid has 60 × 3 = 180 monomers, a *T* = 4 capsid has 60 × 4 = 240 monomers, and so on. Viruses on Earth have been identified with *T* numbers >100 [23].

Thus, in interrogating extra-terrestrial materials for signs of life, a fruitful approach might be to hunt for particles constructed from subunits with numbers matching the *T* numbers. For many environments on Earth, viral particles outnumber cells; if this holds in extraterrestrial environments, hunting for virus-like particles may provide a more abundant target for study than cell-like structures. Such quantification would likely require new technology, but recently reported methods for analysis of single protein molecules might represent a starting point [24–26]. Tracking possible contamination would also be a challenge. Nevertheless, measuring the numbers of subunits in particles from extrasolar sources and finding matches to the *T* numbers could suggest virus-like structure, even without knowing the full composition of the polymer comprising the particle.
